# Random forest model to identify factors associated with anabolic-androgenic steroid use

**DOI:** 10.1186/s13102-021-00257-5

**Published:** 2021-03-23

**Authors:** Zohreh Manoochehri, Majid Barati, Javad Faradmal, Sara Manoochehri

**Affiliations:** 1 Department of Biostatistics, Student Research Committee, Hamadan University of Medical Sciences, Hamadan, Iran; 2 Department of Public Health, School of Health, Autism Spectrum Disorders Research Center, Hamadan University of Medical Sciences, Hamadan, Iran; 3 Modeling of Noncommunicable Diseases Research Center, Department of Biostatistics and Epidemiology, School of Public Health, Hamadan University of Medical Sciences, Shahid Fahmideh Boulevard, Hamadan, Iran

**Keywords:** Random forest, Androgenic-anabolic steroids, Prototype willingness model

## Abstract

**Background:**

One of the types of doping that is commonly used by bodybuilders, is androgenic-anabolic steroids (AAS). The use of AAS besides violating sporting ethics would have serious consequences on physical and mental health statuses. This study aimed to determine the most important factors of using AAS among bodybuilders by prototype willingness model (PWM).

**Methods:**

In this analytical cross-sectional study, 280 male bodybuilders were selected from the bodybuilding clubs in Hamadan city using multistage sampling in 2016. A self-administered questionnaire consisting of demographic information and constructs of the PWM was then used to collect data and random forest model was also applied to analyze the collected data.

**Results:**

Behavioral willingness, attitude, and previous AAS use were found as the most important factors in determining the behavioral intention. Moreover, subjective norms, attitude, BMI, and prototypes were the factors with the greatest effect on predicting behavioral willingness of AAS use. As well, behavioral intention was observed to be more important than behavioral willingness for predicting of AAS use.

**Discussion:**

The obtained results show that the reasoned action path has a greater impact to predict AAS use among bodybuilders compared to social reaction path.

**Supplementary Information:**

The online version contains supplementary material available at 10.1186/s13102-021-00257-5.

## Background

**Fig. 1:**
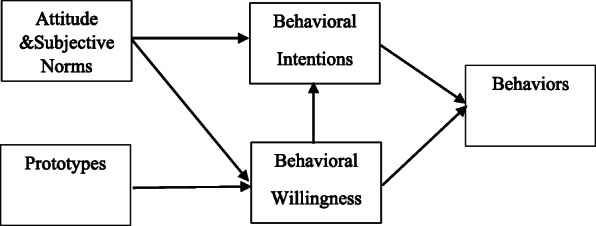
The prototype willingness model. Adapted from Gibbons et al [15].

Androgenic Anabolic Steroids (AAS) are a group of steroids containing natural androgens such as testosterone and testosterone-like industrial substances in terms of structure and function [1]. The history of using these substances for medical purposes goes back to almost 90 years ago. AAS increase muscle size and strength in healthy men [2]. The prevalence rate of these substances is between 1 and 5% worldwide and their usage is more common among men [3]. It is estimated that 2.9 to 4 million people among the US population aged between 13 and 50 years old use these substances, so it can be said that about 1 million people in this population are heavily dependent on them [4]. A questionnaire-based study conducted on elite college athletes who were active in 23 different sports in the United States in 2013 estimated the prevalence rate of steroids around 20% [5]. To the best of the authors’ knowledge, there are no studies investigating the prevalence of AAS use in Iran so far; however, a study in Shiraz found the prevalence rate of AAS among men’s bodybuilders as 39% [6]. Some of the most common motivations for using AAS as reported by bodybuilders in Kermanshah were as follows: Increasing muscle mass, increasing physical strength, dietary supplementation, and making the body more beautiful [7]. Another important reason for AAS use is to be competitive in bodybuilding competitions [8]. The use of these drugs has not been approved for healthy adults in many countries. Correspondingly, in Iran, steroids must be legally prescribed by a physician. In 1975, the International Olympic Committee included steroids in the list of the banned drugs [9]; however, many athletes continued to use it. Besides violates sports ethics, AAS use also has some serious consequences on physical and mental health statuses, including cardiovascular problems; renal complications; thyroid disorder; tendon and ligament rupture; and major mood disorders such as aggression and violence and even death [10, 11]. Since having a muscular and beautiful body, along with the desire to get into the sports position have become important social indicators, young people are increasingly willing to use these substances [7]. Health-centered educational interventions can partially reduce the tendency to use these drugs; and therefore, these interventions reduce substance abuse [12]. However, some experts believe that one of the reasons for the failure of educational programs is the lack of paying enough attention to psychosocial factors such as having the ability to reject the offer for use and self-control [13]. In this regard, to explain the phenomenon of substance use, different theories have been proposed. Although these theories are not specific to AAS, they provide a useful understanding on AAS use. Accordingly, one of these theories is the prototype willingness model (PWM) [14]. According to PWM, there are two basic paths to detect high-risk behavior as follows: the reasoned action path and social reaction path. The reasoned action path refers to an analytical and argumentative process including structures of attitudes, subjective norms, and behavioral intentions. The social reaction path is based on imagination, which includes an exploratory process explaining the behavior of individuals with no prior intention. Moreover, this path includes structures of prototypes and behavioral willingness (Fig. 1) [15]. In PWM, having intention to perform a behavior is determined by the following two factors: attitude and subjective norms. Attitude is a positive or negative evaluation of a person regarding performing a behavior and subjective norms that refer to the social pressure perceived by the individual either to do or not to do the intended behavior. In other words, subjective norms are beliefs that most of people during their life think about that they should do a behavior or should not do it. For example, the best friend of an athlete or his coach plays a decisive role in the case of steroid use. Additionally, behavioral willingness can be predicted by prototypes and subjective norms constructs as such these constructs are mental images of the subjects with high-risk behaviors [14]. Prototypes are people’s mental images of a person from their age who is involved in a certain high-risk behavior like being a typical smoker. A more positive image leads to having a greater tendency towards smoking [16]. Each component of the PWM structure is evaluated with several questions that are described in the Methods section of this study, which can be very helpful in understanding this model. Based on the results of several studies, the PWM has shown a good performance in predicting risky behaviors such as smoking [17, 18]. Therefore, many researchers have focused on identifying the risk factors for substance abuse in order to develop an effective preventive strategy in this field [19, 20].

To describe the relationship between risk factors and response, classical statistical models such as regression and path analysis, can be used. Classical statistical models are very welcomed in terms of interpretability, but this interpretation’s basis is the strong hypothesis of “knowing the form of the relationship” that must be determined by the analyzer. If the form of the choice relationship between the response and the risk factors is not correctly determined, the results would mislead the researcher. To this end, “machine learning” methods have been developed whose main purpose is to overcome this problem. In these methods, the relationship is determined based on the functional form of data itself [21, 22]. Of note, there are various regression and classification methods in the domain of machine learning, among which the tree-based regression model has been more preferred for its high-order nonlinear models as well as its high interpretability [23]. Moreover, the random forest model based on the development of several decision trees was found to be able to predict the response and determine the importance of each variable more accurately [24, 25]. Notably, random forest model has not been used to determine the relationship between different factors and AAS use, so far. Therefore, this study aimed to apply random forest model to determine the importance factors that are effective on (a) Behavioral intention; (b) Behavioral willingness, and (c) AAS use, among the bodybuilders of Hamadan city.

## Methods

### Study setting, population, and sampling method

In this secondary analytical cross-sectional study, the population were male bodybuilders referred to bodybuilding clubs of Hamadan city, the capital of Hamadan province, west of Iran. The inclusion criteria were age range 15–45 years old and having more than 6 months history of activity in a bodybuilding club.

The sampling method used in the initial study is briefly expressed hereafter and more information can be found in the initial study [12]. The participants were selected using a multistage sampling. The whole population was classified into three geographical zones and 5 clubs from the 1st, 3 clubs from the 2nd, and 2 clubs from the last zones were randomly selected. Twenty-eight athletes were selected from each club using simple random sampling. Finally, 280 subjects were included in the study.

### Data collection

In the present study, a self- administered questionnaire consisting of 2 sections was used to collect data. The first part of the questionnaire consisted of demographic information, including age; level of education; marital status; body mass index (BMI); and history of sport club, tobacco smoking, alcohol consumption, AAS usage by the best friend, AAS usage by coach, and nutrition supplement use. The second part was about the constructs of the PWM. The face and content validity of the questionnaire were both assessed by a panel of experts using 10 health education experts’ opinions. For this purpose, content validity ratio (CVR) and content validity index (CVI) for the questions were extracted and by considering the values of the Lawshe table (CVR > 0.62 and CVI > 0.79), the questions were then reviewed and finally corrected. To evaluate the reliability of the questionnaire, a preliminary study was performed on 32 athletes and the internal consistency of the questions was reviewed using Cronbach’s alpha and then confirmed [26].

The PWM consisting of the following 6 constructs:

- Positive attitude towards AAS use: Included 6 specific questions (e.g., “Taking anabolic steroids helps me in having a stronger body”) with a 5-point Likert scale ranged from 1 (strongly disagree) to 5 (strongly agree), in a way that higher score indicates a more positive attitude towards anabolic steroids use. Cronbach’s alpha of this structure was estimated as 0.798 in the pilot study [26].
- Subjective norms: consisted of 4 questions related to the best friend, and coaches, with a 5-point Likert ranged from 1 (not at all) to 5 (very). One example of these questions is “If I want to use anabolic steroids, my coach will approve it”. Accordingly, in this scale, higher scores indicate higher subjective norms that encourage the use of anabolic steroids. Cronbach’s alpha of this structure was estimated as 0.701 in the pilot study [26].
- Prototype from AAS use: Bodybuilders’ images of substance use is expressed in the following sentence: “Imagine having a bodybuilding friend of your age who regularly take anabolic steroids. In your opinion, each one of the following traits is more proper to describe him?” .It consisted of 10 items with a 5-point Likert ranged from 1 (not at all) to 5 (very). These ten items were as follows: happiness, proudness, kindness, strong, nervousness, high sexual desire, violence, attractiveness, bully, illiteracy. Higher score on this questionnaire indicates that one’s mental conception is positive for peers taking AAS. Cronbach’s alpha of this structure was estimated as 0.902 in the pilot study [26].
- Behavioral intention of AAS use: It consisted of 4 questions with a 5-point Likert. For example, one of these questions is “Do you want to use anabolic steroids for the next 6 months to improve your athletic performance?” Higher scores in this questionnaire indicate the individual tendency towards taking AAS. Cronbach’s alpha of this structure was estimated as 0.770 in the pilot study [26].
- Behavioral willingness of AAS use: This structure began by describing a hypothetical scenario as follows: “Imagine you are in the midst of your bodybuilding friends and there is anabolic steroids available. If your closest bodybuilding friend suggests you the use of anabolic steroids, how likely are you to do one of the following options?” The 4 items to respond to this scenario are the followings: a) you take it and use it, b) you only use it once or twice, c) you say no thanks and you continue your activity in the club, d) you leave your gym and then sign up for a new club. Each item consists of 5-choice spectrum with a minimum score of 4 and a maximum of 20. A higher score on this questionnaire indicates higher willingness for AAS use. Cronbach’s alpha of this structure was estimated as 0.729 in the pilot study [26].
- AAS use behavior: this construct was measured by the following two questions: (1) Are you currently using AAS? And (2) have you ever used AAS in your lifetime?. The answers to these questions consist of two categories as yes or no.

### Statistical methods and software

The sample was described using appropriate descriptive statistics. To compare quantitative variables in the two groups (those with and those without currently AAS use), independent samples t-test was used and for qualitative variables, chi squared test was applied. To determine the importance of the effective factors on the outcomes (behavioral intention, behavioral willingness, and AAS use), random forest model was separately applied. In order to evaluate the performance of the random forest model, we divided the obtained data into two parts, in a way that 80% of the data was used for training and 20% others were used for testing.

#### Decision tree & random forest models

A random forest is an ensemble learning method, which consists of many decision trees. A decision tree is a simple and robust method used to classify a dataset into distinct and homogeneous categories. In addition, the decision tree algorithm has a non-rotating tree-like graph represented by a set of questions. Usually, each question is represented with a variable. A decision tree graph consists of the following three main components: root, inner node, and outer node (leaf). The process of developing a tree is such that all people firstly fall into the root node. Thereafter, based on a certain feature, individuals are divided into two groups and each is then placed in a new node. Subsequently, each new node, like the root node, is splitted into other nodes to achieve a level of homogeneity in the response of the individuals in each node eventually. Notably, these nodes are also called leaf nodes. The selection of the variable to divide the individuals in a node into two parts is performed based on its relationship with the response variable, so that the variable that creates the most homogeneity in response in the resulting nodes is selected at each step [27]. Normally, decision tree models are trained at two stages, namely partitioning in a two-step process. In the first step, binary recursive partitioning is used to construct the tree structure, which was mentioned earlier in the two paragraphs above. Secondly, pruning is done, in order to remove leaves that do not help in improving tree prediction in a new set of data [23]. In the random forest, a large number of trees are constructed based on random subsets of data and prediction is then made based on the average prediction of each tree [25].

#### Software

SPSS software version 24 was used to describe the data and also to perform independent samples t-test, chi-square tests, and one-way analysis of variance. To develop the random forest using R3.6.2, randomForest [28] package was used.

## Results

Among a total of 280 bodybuilders, 35 (12.5%) subjects were the current AAS users and 245 (87.5%) subjects were not the current AAS users (81.6% of them have never used it). The mean (SD) age of the participants was 25.21 (6.35) years old.

Table 1 presents some demographical features of bodybuilders participating in this study in terms of the current use of AAS. According to the results shown in Table 1, compared to those who were not the current AAS users, the subjects with AAS use had more history of sport club and were more likely to use alcohol and nutritional supplement (*P*-value < 0.05). Additionally, there was a statistically significant difference between user and non-user of AAS in terms of AAS use by coach and by the best athlete friend (*P*-value < 0.05).

**Table 1:** Demographical feature and status of activity in club of participants in terms of currently AAS use

**Quantitative features**	**Current AAS use (*n*** **= 35)**	**No current AAS use (*n*** **= 245)**	***P*-value**
	**Mean (SD)**	**Mean (SD)**	
**Age**	24.51 (4.95)	25.31 (6.53)	0.491
**Weight**	79.40 (14.51)	78.30 (12.70)	0.638
**Body mass index (BMI)**	24.94 (4.70)	24.74 (3.52)	0.766
**History of sport club (month)**	66.00 (65.56)	44.37 (54.00)	0.032*
**Qualitative features**	**N (%)**	**N (%)**	***P*-value**
**Marriage status**			
**Single**	27 (77.1)	196 (80.0)	0.659
**Married**	8 (22.9)	49 (20.0)	
**Level of education**			
**Lower than diploma**	7 (20.0)	26 (10.6)	0.316
**Diploma**	16 (45.7)	103 (42.0)	
**Academic**	12 (34.3)	116 (47.3)	
**Titleholder in bodybuilding**			
**No**	14 (40)	128 (52.2)	0.370
**Regional**	16 (45.7)	97 (39.6)	
**National**	5 (14.3)	20 (8.2)	
**Alcohol use**			
**Always**	4 (2.4)	6 (1.9)	0.009*
**Sometimes**	11 (31.4)	54 (22.0)	
**Never**	20 (57.1)	185 (75.5)	
**Tobacco smoking**			
**Always**	4 (11.4)	10 (4.1)	0.127
**Sometimes**	5 (14.3)	26 (10.6)	
**Never**	26 (74.3)	209 (85.3)	
**Previous AAS use**			
**Always**	7 (20.0)	4 (1.6)	< 0.001*
**Sometimes**	24 (68.6)	41 (16.7)	
**Never**	4 (11.4)	200 (81.6)	
**AAS usage by the Coach**			
**Always**	11 (31.4)	39 (15.9)	0.042*
**Sometimes**	12 (34.3)	76 (31)	
**Never**	12 (34.3)	130 (53.1)	
**AAS usage by the best friend**			
**Always**	11 (31.4)	37 (15.1)	< 0.001*
**Sometimes**	19 (54.3)	54 (22)	
**Never**	5 (14.3)	154 (62.9)	
**Nutritional supplement use**			
**Always**	13 (37.1)	36 (14.7)	0.002*
**Sometimes**	17 (48.6)	128 (52.2)	
**Never**	5 (14.3)	81 (33.1)	

Sign * indicates significant test in level 0.05

Table 2 reports the relationship among PWM structures. As shown in this table, among these structures, the highest correlation was found to be between behavioral intention score and behavioral willingness score for AAS use (*r* = 0.574, *P*-value < 0.001). On the other hand, the least linear relationship was observed between prototypes score and behavioral willingness score (*r* = − 0.029, *P*-value > 0.05).

**Table 2:** Correlation between different structures of PWM for ASS use

Variables	1	2	3	4	5	Mean (SD)
**1. Attitude**	1.000	0.312*	0.150*	0.514*	0.314*	17.01 (5.15)
**2. Subjective norms**		1.000	0.144*	0.267*	0.338*	9.52 (3.75)
**3. Prototypes**			1.000	0.085	−0.029	29.34 (8.67)
**4. Behavioral intention**				1.000	0.574*	8.16 (3.91)
**5. Behavioral willingness**					1.000	7.36 (3.24)

Sign * indicates significant test in level 0.05

The finding presented in Tables 1 and 2 are crude and reported without considering the simultaneous effects and possible interactions among various features. In this situation, Random forest model could be used to develop a predictive model and then extract the importance of each feature, in order to predict AAS use. As it was stated earlier in pevious section, a random forest is consisted of many individual trees, which were counted as 500 in this study.

**Fig. 2:**
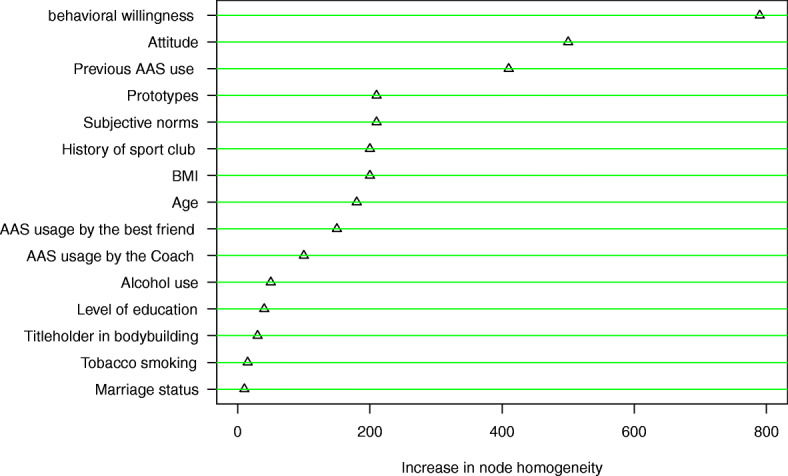
The variables importance based on random forest for prediction of behavioral Intention of AAS use

Based on the results of fitted random forest, the most important feature in determining the behavioral intention for AAS use was found to be behavioral willingness in terms of the homogeneity in subgroups. Moreover, attitude, previous AAS use, prototypes, subjective norms, history of sport club, and BMI were found as the next most important factors in predicting the behavioral intention of AAS use. The order of the factors from the most important in predicting the behavioral intention of AAS use to the least important one is reported in Fig. 2.

**Fig. 3:**
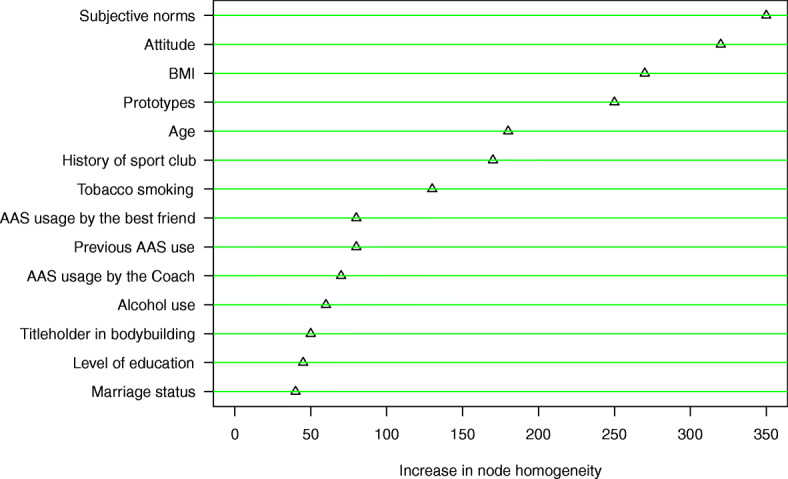
The variables importance based on random forest for prediction of behavioral willingness of AAS use

Furthermore, the order of the factors from the most important in predicting the behavioral willingness of AAS use to the least important one is reported in Fig. 3. As shown in Fig. 3, subjective norms, attitude, BMI and prototypes were found as the factors with the greatest effect on predicting behavioral willingness of AAS use.

**Fig. 4:**
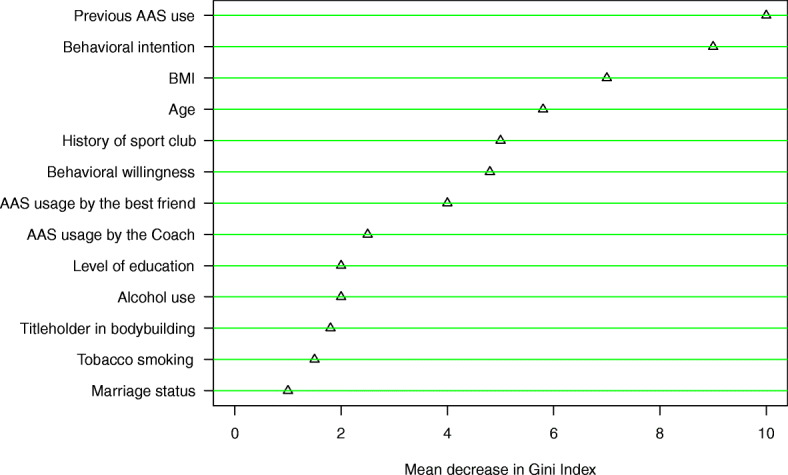
The variables importance based on all factors by random forest for prediction of AAS use

Based on the results of the random forest model fitted based on the all factors, which are presented in Fig. 4, previous AAS use, behavioral intention, BMI, age, history of sport club, and behavioral willingness could predict AAS use well.

**Fig. 5:**
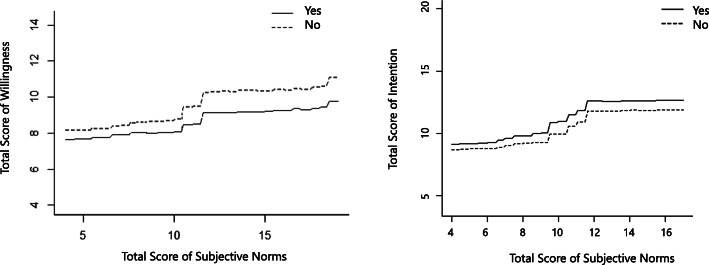
Relationship between behavioral willingness and subjective norms (left), Relationship between behavioral intention and behavioral willingness (Right) For subjects with (continuous line) and without currently AAS use (dashed line)

A part of the outcome of random forest is presented in Fig. 5. As indicated in this figure, both subjective norms and behavioral intention were observed to have a direct association with willingness for AAS use. As well, in the right panel, the subjects with AAS use have higher behavioral intention score.

## Discussion

This study aimed to investigate the effective factors on AAS use among bodybuilders in Hamadan. To do this, PWM was used. The importance of the methodin the present study was the use of random forest data mining model to investigate the effect of different factors on AAS use. Of note, one of the important features of the random forest model is the determination of the functional form of the relationship between the predictors and the response through the data itself. In other words, the functional form of the relationship between different factors and AAS use is not selected by the analyst, but the data determine this functional form. Therefore, this model is able to take the complex relationships between different factors and response into account and then to provide more accurate predictions. However, based on our search in different databases, no study has been performed using PWM and random forest model to predict the factors affecting AAS use so far.

According to the results of the univariate analysis (Table 1), history of sport club, alcohol use, usage by coaches, usage by the best athlete friend, and nutritional supplement use were more likely in AAS users compared to the other subjects, so this difference was found to be statistically significant (*P*-value < 0.05). However, the two groups did not differ significantly in terms of demographic variables such as age, level of education, and marital status (*P*-value > 0.05). In this study, 27.1% of bodybuilders reported a history of anabolic steroid use, which is consistent with a similar study performed in this field [29]. However, in the present study, the rate of steroid use was higher than 4.3% that was reported in the study by Ghobain et al. [30] and it was less than 64% that was reported in the study by Bijeh et al. [31]. This discrepancy may be due to differences in the target community as well as differences in the geographic areas studied. For example, the target community of Ghobain’s study was all Saudi athletes, but if these studies have been performed only on professional bodybuilders, it can be said that the prevalence of anabolic steroid use would increase [32].

Based on the results, the highest correlation was obtained among behavioral intention and behavioral willingness (*r* = 0.574) and attitude (*r* = 0.514). Thus, people with higher behavioral willingness scores also had higher behavioral intention and attitude scores. The results of the random forest analysis confirmed the above-mentioned results (Fig. 2), as behavioral willingness and attitude have the greatest effect on predicting behavioral intention. In the study by Patiro et al., there was a direct relationship between attitude and behavioral intention of AAS use, which is consistent with the results of the present study [33]. As well, in the study by Abedini et al., attitude, subjective norms, and behavioral willingness were reported as the predictors of intention to use hookah among students [17].

Notably, the analysis of the social reaction path in PWM showed that subjective norms, attitude, and positive prototypes related to AAS users are associated with willingness to AAS use. This result is consistent with the results of a study in which the attitude and prototypes were found as the strong predictors of willingness to doping [34]. Moreover, the results of a similar study showed that attitude, subjective norms, and prototypes were the factors that predicted willingness to smoking among adolescents in Hamadan city. Correspondingly, these factors predicted 43% of willingness variation that in the meantime, the role of subjective norms was more prominent than attitude and prototypes [35]. Various studies conducted on substance abuse prevention showed that training some life skills such as problem solving and decision-making skills increase cognitive-coping skills, which consequently reduces the tendency of individuals to use a variety of illicit substances. In this regard, other studies have pointed out the resistance skills’ training against insistence of peer such as the skill of saying “no” [36].

The results of the present study show that reasoned action path is a better predictor for substance use compared to social reaction path. By comparing the order of importance of intention and willingness in the prediction of AAS use, the results showed that behavioral intention is a better predictor of AAS use (Fig. 4). In other words, the decision-making process for anabolic steroids use is mostly based on people’s previous intentions. In this regard, various studies have been performed to confirm the effect of behavioral intention on predicting and occurring high-risk behaviors [37–39]. However, the results of a study by Barati et al. showed that behavioral willingness was a better predictor of smoking behavior compared to behavioral intention and these two structures predicted a total of 43% of behavior variation [35]. In other studies related to the PWM, the behavioral willingness structure was shown to have a higher predictive ability in comparison with behavioral intention that is not consistent with the findings of the present study [40, 41]. Age and previous experiences of individuals may explain the inconsistency between the results of the present study those of the other studies. Usually, by aging, people’s life experiences also increase, so they make more rational decisions [42]. The mean age of the participants in the present study was 25.21 years old, while in other studies, participants were mostly adolescents. Evidence suggests that the relationship between intention and behavior is weaker in adolescents compared to other groups of individuals [14]. On the other hand, some similar studies have described the experience as the reason for this problem and pointed to the weakness of the relationship between intention and behavior in less experienced people [43]. As can be seen from the results of random forest (Fig. 4), age is one of the most important factors of predicting AAS use.

## Limitations

One of the limitations of this study was the poor cooperation of some bodybuilder participants in filling the questionnaire. Besides, this study was performed only on male bodybuilders; therefore, it is suggested to conduct comprehensive comparison between male and female bodybuilders as well as athletes of other sporting disciplines such as wrestling, weightlifting, and gymnastics in future studies. We only had access to already collected data, as a result, variables such as performance and image-enhancing drugs (PIEDs) that were not collected in the primary study, could not be included in the analysis. Furthermore, make a comparison between (recent) users of AAS and non-users might provided useful information. To do this comparison, it need to exclude recent users of AAS who are not current users. This would reduce the sample size and the generalizability of the study. Therefore, it suggested that these issues be addressed in a future study.

## Conclusion

The results indicate the importance of reasoned action path in PWM compared to social reaction path. Therefore, it is recommended to implement educational programs using the PWM and put the emphasis on subjective norms and behavioral intention to facilitate the prevention of AAs use.

## Supplementary Information

**Additional file 1.**

## Data Availability

The datasets used and/or analyzed during the current study are available from the corresponding author on reasonable request.
