# Supplementary material for: Random forest model to identify factors associated with anabolic-androgenic steroid use
Source: BMC Sports Sci Med Rehabil. 2021 Mar 23;13:30. doi: 10.1186/s13102-021-00257-5 (PMC7988984; doi:10.1186/s13102-021-00257-5)
Supplement: Supplementary file 1 — Additional file 1. [file 13102_2021_257_MOESM1_ESM.docx]

1. Age: ……years
2. Weight:……….. kg
3. Body mass index (BMI): …………$(\frac{kg}{{cm}^{2}})$
4. Degree of education: Lower than diploma □ Diploma □ Academic □
5. Marriage status: Single □ Married □
6. History of sport club : …….(month)
7. Do you have a titleholder in bodybuilding? No □ Regional □ National □
8. Do you drink alcohol? Always □ Sometimes□ Never□
9. Do you tobacco smoke? Always □ Sometimes□ Never□
10. Do you use nutritional supplement (other than anabolic steroids)? Always □ Sometimes□ Never□
11. Do your coaches use anabolic steroids? Always □ Sometimes□ Never□
12. Does your best bodybuilder friend use anabolic steroids? Always □ Sometimes□ Never□
13. Have you used anabolic steroids in the past? Always □ Sometimes□ Never□

1.Completely agree 2. Agree 3. No opinion 4. Disagree 5. Completely disagree

| **5** | **4** | **3** | **2** | **1** | **Attitude** |
| --- | --- | --- | --- | --- | --- |
|  |  |  |  |  | 1. The use of anabolic steroids helps me to have a stronger body. |
|  |  |  |  |  | 2. The use of anabolic steroids helps to perform better in competitions. |
|  |  |  |  |  | 3. I think anabolic steroids can cause dangerous diseases. |
|  |  |  |  |  | 4. The use of anabolic steroids is a great way to build muscle. |
|  |  |  |  |  | 5. The use of anabolic steroids makes me to be noticeable by others. |
|  |  |  |  |  | 6. The use of anabolic steroids helps me to reach my athletic goals more easily. |

1.Too many 2. Many 3. Some extent 4. Low 5. Not at all

| **5** | **4** | **3** | | **2** | **1** | **Subjective Norms** |
| --- | --- | --- | --- | --- | --- | --- |
|  |  |  | |  |  | 1. How many members of your club use anabolic steroids? |
|  |  |  |  | |  | 2. If I use anabolic steroids, my coach will approve it. |
|  |  |  |  | |  | 3. If I use anabolic steroids, my clubmate will approve it. |
|  |  |  |  | |  | 4. If I use anabolic steroids, my best friend will approve it. |

“Imagine a bodybuilding friend of your age regularly taking anabolic steroids. In your opinion each of the following traits is appropriate to describe his?”

1.Too many 2. Many 3. Some extent 4. Low 5. Never

| **5** | **4** | **3** | | **2** | **1** | **Prototypes** |
| --- | --- | --- | --- | --- | --- | --- |
|  |  |  | |  |  | 1. happiness |
|  |  |  |  | |  | 2. proudness |
|  |  |  |  | |  | 3. kindness |
|  |  |  |  | |  | 4. strong |
|  |  |  |  | |  | 5. nervousness |
|  |  |  |  | |  | 6. high sexual desire |
|  |  |  |  | |  | 7. violence |
|  |  |  |  | |  | 8. attractiveness |
|  |  |  |  | |  | 9. bully |
|  |  |  |  | |  | 10. illiteracy |

1.Too many 2. Many 3. Some extent 4. Low 5. Never

| **5** | **4** | **3** | | **2** | **1** | **behavioral intention** |
| --- | --- | --- | --- | --- | --- | --- |
|  |  |  | |  |  | 1. I want to use anabolic steroids for the next 6 months to improve my athletic performance. |
|  |  |  |  | |  | 2. I want to use anabolic steroids for the next 1 month to improve my athletic performance. |
|  |  |  |  | |  | 3. I would like to recommend anabolic steroids to my friends. |
|  |  |  |  | |  | 4. I intend to continue bodybuilding only with exercise and without the use of anabolic steroids. |

Imagine you are in the midst of your bodybuilding friends and there is anabolic steroids available. If your closest bodybuilding friend suggests you the use of anabolic steroids, how likely are you to do one of the following options?

1. Never 2. Low 3. Some extent 4. Many 5. Too many

| **5** | **4** | **3** | | **2** | **1** | **behavioral willingness** |
| --- | --- | --- | --- | --- | --- | --- |
|  |  |  | |  |  | a) You take it and use it |
|  |  |  |  | |  | b) You only use it once or twice |
|  |  |  |  | |  | c) You say no thanks and you continue your activity in the club |
|  |  |  |  | |  | d) You leave your gym and then sign up for a new club |

| **behavioral** |
| --- |
| 1. Are you currently using AAS? Yes  No |
| 2. have you ever used AAS in your lifetime? Yes □ No□ |
